# Grit and Life Satisfaction Among College Students During the Recurrent Outbreak of COVID-19 in China: The Mediating Role of Depression and the Moderating Role of Stressful Life Events

**DOI:** 10.3389/fpubh.2022.895510

**Published:** 2022-05-18

**Authors:** Haidong Liu, Zhijun Yu, Baojuan Ye, Qiang Yang

**Affiliations:** ^1^School of Psychology, Jiangxi Normal University, Nanchang, China; ^2^School of Education, Jiangxi Normal University, Nanchang, China

**Keywords:** grit, life satisfaction, depression, stressful life events, COVID-19, Chinese college students

## Abstract

The global recurrent outbreak of COVID-19 has brought immense psychological distress to those affected. We conducted this study to explore the relationship among grit, stressful life events, depression, and life satisfaction in college students during the recurrent outbreak of COVID-19. According to the properties of the bias-corrected bootstrap method, we surveyed 888 college students, with an average age of 20.84 (*SD* = 1.57) years. Participants completed questionnaires regarding grit, depression, stressful life events, and life satisfaction. The results showed that 1) grit was correlated with life satisfaction (*r* = 0.426, *p* < 0.001); 2) depression mediated the relationship between grit and life satisfaction [indirect effect = 0.0292, *SE* = 0.009, 95% *CI*_boot_ = (0.135, 0.500)]; 3) The relationship between grit and depression was moderated by stressful life events (β = 0.107, *SE* = 0.028, *p* < 0.001, 95% *CI* [0.053, 0.161]). The association between grit and depression became weaker for college students with high stressful life events. The results indicated that concerned about depression and stressful life events may be the main targets for improving life satisfaction among college students during the recurrent outbreak of COVID-19.

## Introduction

Life satisfaction was first described in the psychological literature by Shin (1), was referred to as a cognitive assessment of an individual's entire life. Life satisfaction is an important indicator to measure an individual's living situation, and it has received more and more attention. In addition, Proctor et al. (2) found that life satisfaction can affect an individual's future psychological state, and has important implications for an individual's physical and mental development. Previous researches have shown that higher life satisfaction predicts an individual's future mental state, and also improving college students' academic performance, academic well-being, and lowering student psychology health risks; lower life satisfaction predicts mental dysfunction (3). Therefore, improving life satisfaction is critical for college students to adapt to school and grow up healthily. College students are at a stage where they are not clear about their future plans and constantly adjust their life goals, so their life satisfaction is highly susceptible and unstable (4). In that case, any events they experience in their lives can easily affect their life satisfaction. Since the outbreak of COVID-19 in 2020, it has been exerting tremendous influence not only on the physical behavior of individuals but also on their mental health (5). At present, tens of thousands of people around the world are infected with COVID-19. Study suggests that people who are quarantined during the COVID-19 experience anxiety, anger, confusion, and stress which may affect people's life satisfaction (6). The perception of stress, social adaptation to the COVID-19, and a series of epidemic control measures (blocking schools, maintaining social distance, prohibiting large student gatherings, etc.) during the COVID-19 epidemic may lead to a decline in the life satisfaction of college students (7–9). So, it is important to explore the factors affecting individuals' life satisfaction during the recurrent outbreak of COVID-19. Helping individuals improve their life satisfaction is important.

### Grit and Life Satisfaction

Because life satisfaction is so important to an individual's mental health, researchers are increasingly focusing on the factors that influence individual life satisfaction. People generally believe that individual traits explain why some are more satisfied with their lives than others in the same circumstances (10). Character strengths theory points out that character strengths is vital for improving individual life satisfaction and obtaining happiness (11). Grit, as a personal trait, is sustained enthusiasm and persistence for long-term goals (12), and has been one of the research hotspots in psychology in recent years. Research shows that grit is related to individual academic achievement, career success, and physical health (13, 14). Life satisfaction is deeply affected by individual life purpose (15). The purpose of life often needs to be achieved through the pursuit of meaningful goals, and grit plays a vital role in this process (16). People with high grit may not have high IQ, but have high perseverance and enthusiasm for long-term goals (10). Individuals with high level of grit are more likely to be inspired to achieve the goal of life and pursue the meaning of life (17). Therefore, grit is of great significance to the growth of college students and can encourage college students to pursue and achieve their life goals through efforts when they are in adversity, and improve their life satisfaction in the process. Previous studies have shown that grit is also positively correlated with life satisfaction, and individual with higher grit also have higher life satisfaction (18). The COVID-19 changes individual's lifestyles and has an impact on people's life satisfaction. However, grit can help an individual maintain mental health in the adaptation to social changes. During the period of the outbreak of COVID-19, the mental health of individuals with high grit is higher than that of individuals with lower grit (19). Therefore, during the epidemic period, the life satisfaction of Chinese college students with high grit is also higher, while the life satisfaction of college students with low grit quality is lower. This study will test the relationship between grit and college students' life satisfaction during the recurrent outbreak of COVID-19 and reveal the underlying mechanism.

### The Mediating Role of Depression in the Relation Between Grit and Life Satisfaction

Although past research has accumulated lots of knowledge about the relation between grit and life satisfaction (18, 20), some questions have not been fully discussed. For example, though previous theoretical studies have shown that grit may be related to life satisfaction, few studies empirically test the potential mechanism between them. Depression may mediates the path of grit relating to life satisfaction. Depression is a key index to diagnose the level of individual mental health and usually refers to the continuous negative emotional experience in individuals' lives, such as depression, anxiety, sadness, pain, etc (21). Individuals with high depression usually have sleep disorders, loss of appetite, self-mutilation, suicide, other behaviors (22). Depression has become one of the main diseases endangering human health. Depression in COVID-19 poses a serious threat to the mental development of young people, especially college students (23). In China, a study shows that depression due to COVID-19 is prevalent among University students (24), and the pooled prevalence estimate of clinically elevated depressive symptoms for adolescents during the COVID-19 pandemic is 30.6% (25). Grit, as an important psychological trait, helps an individual maintain mental health in the adaptation to social changes, and is positively correlated with well-being and life satisfaction (16). From the perspective of resource theory (26), the grit of college students is an important positive psychological resource for individuals to deal with life pressure. Individuals with a higher level of grit are more likely to see difficulties and stress in life as an inevitable part of the struggle, which makes individuals with a higher level of grit be less likely to be depressed (26). The study has shown that the occurrence of depression is inversely related to the quality of life and that more severe depression is associated with a decline in quality of life (27). The decline in life quality will lead to decline in individuals' life satisfaction, and previous studies have found a significant negative correlation between depression and life satisfaction (28). Therefore, this study believes that depression plays a mediating role in the process that college students' grit positively affects life satisfaction.

### The Moderating Role of Stressful Life Events

Grit may affect depression, but not for everyone, so the process by which perseverance affects depression may be influenced by other factors. Previous studies on grit and depression only focused on the effect of grit on depression (29–31), and did not integrate grit and environmental characteristics into consideration of their interaction effects on depression. Specifically, the organism-environment interaction model holds that individuals and the environment are complex systems in which the elements do not act independently but depend on each other (32). That is, the role of individual factors (such as grit) may vary with environmental factors (such as stressful life events). As the most basic environmental factor in an individual living environment, stressful life events have an impact on the growth of college students. Stressful life events refer to some negative life events that people may encounter in daily life and bring pressure to individuals, which can have a negative impact on individuals' mental health (33), and it is of great significance to the development of individual psychology and behavior. A previous study has pointed out that if faced with fewer stressful life events, individuals can cope normally, but if faced with more stressful life events, the individual's psychological adjustment mechanism will be damaged, resulting in some psychological problems (34). According to the above reaches, both individuals with higher grit and low grit may have different effects on depression when faced with less stressful life events and more stressful life events. O'neal et al. (35) have shown that stressful life events negatively affect an individual's grit. Therefore, stressful life events may moderate the process of grit affects depression. According to the diathesis-stress interaction theory (36), depression stems from stress in life, and stress in life has a positive predictive effect on depression. So, stressful life events are the risk factors for depression among college students during the recurrent outbreak of COVID-19. Studies show that during COVID-19 the pressure of life on college students has increased, college students face more stressful life events such as pressure from study, employment, interpersonal relationship, and so on (37).

According to the organism-environment interaction model (32), stressful life events and grit may interact with depression of college students. According to the protective-reactive model (37), the effect of one protective factor is greater when the risk factor is higher. The relationship between grit and depression should be stronger for individuals experiencing higher stressful life events than those experiencing lower stressful life events. Based on this, we speculate that during the recurrent outbreak of COVID-19, stressful life events as a risk factor moderate the effect of grit on the depression of college students.

### The Present Study

**Figure 1:**
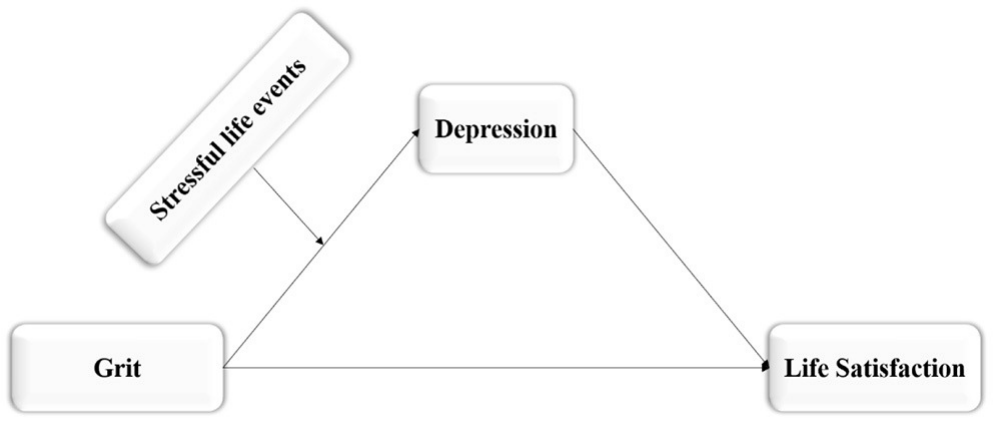
The proposed moderated mediation model.

This study constructed a moderated mediation model (see Figure 1) to test the mediating effect of depression and the moderating effect of stressful life events. Based on existing research conclusions and theories, this study puts forward 2 specific hypotheses:

Hypothesis 1: depression mediates the relationship between grit and life satisfaction.

Hypothesis 2: stressful life events moderate the association between grit and depression.

## Method

### Participants

A total of 907 college students from China were surveyed and all filled out the questionnaire, and 888 participants were left after deleting invalid participants (e.g, < 100 s to complete questionnaires). Therefore, the valid response rate of this study was 97.91%. Among them, there were 381 man (42.9%) and 507 women (57.1%), aged between 17 and 25(*M* = 20.84, *SD* = 1.57). There were 306 (42.2%) rural residents and 647 (57.8%) urban residents.11.3% were 1st years, 30% were 2nd years, 29.7% were 3rd years, 21.7% were 4th years.

### Measures

#### Grit Scale

College students' grit was assessed with new version of the grit scale-Oviedo Grit Scale developed by Postigo et al. (38), which was used to measure grit. This scale is one-dimensional, with 10 items (e.g. “*I spend as much time and energy as I can on reaching my goals*”). Participants rate each item on a 5-point scale (1 = strongly disagree to 5 = strongly agree). Individual with higher total scores indicating higher levels of grit. In this study, these 10 items were forward and back-translated by Chinese professors who were fluent in both Chinese and English. And we did some slight changes to make sure the items could be applied to typical Chinese cultures. The scale had good validity in this study and was in line with various psychometrics standards. The Cronbach's alpha of the scale in this study was 0.891. Validity information of Oviedo Grit Scale was CFI = 0.973, TLI = 0.965, RMSEA = 0.056, SRMR = 0.026.

#### Stressful Life Events Scale

Stressful life events experienced by the participants were assessed with the Stressful Life Events Scale (39) which consists of 16 items (eg, “*falling behind in study*”). Each item represented a stressful event and participants reported whether or not these stressful events had occurred in the past year. Each item was rated on a 6-point scale (0 = did not occur to 5 = occurred and extremely stressful). The average score for each of the 16 items was calculated. The higher the score, the greater the number of stressful life events they experienced. The Cronbach's alpha of the scale in this study was 0.915.

#### Depression Scale

Depression in this study was assessed with the Patient Health Questionnaire Depression Self-Rating Scale (40). Bian et al. (41) revised the scale in Chinese. The scale is a depression screening tool based on nine symptoms of depression in the Diagnostic and Statistical Manual of Mental Disorders, Edition IV(DSM-IV). Compared with other depression scales commonly used, it has the advantages of fewer items and is easy to understand. There are nine items(eg, “*Feeling down depressed or hopeless*”), and each item was rated on a 4-point scale (0 = not at all to 3 = almost every day), with higher total scores indicating higher levels of depression. The Cronbach's alpha of the scale in this study was 0.922.

#### Life Satisfaction Scale

The Chinese Version of the Life Satisfaction Scale (42), adapted from the Life Satisfaction Scale (43), was used to evaluate college students' life satisfaction. This scale consists of 5 items (e.g., “*In most ways my life is close to ideal*”). Participants rated their life satisfaction on a 7-point Likert scale (1 = strongly disagree to 7 = strongly agree). Higher total scores indicated higher level of life satisfaction. The Cronbach's alpha of the scale in this study was 0.888.

### Procedure

The study was approved by the ethics committee of the first author's University. In this study, participants over the age of 18 provided informed consent, and participants under the age of 18 obtained the consent of their legal guardians. Because of the social distancing order issued by the government during the recurrent outbreak of COVID-19. The survey was hosted on Wenjuan Web (Shanghai Zhongyan International Science and Technology, Shanghai) from December 18 to December 28, 2021. In this study, all responses were anonymous. There was no compensation for participating in this study, and the participants participated entirely voluntarily.

### Analytical Strategy

First of all, SPSS26.0 were used to calculated the descriptive statistics for the study variables, and then their correlation between the study variables were calculated. Next, we tested the mediating effect of grit by using the PROCESS (Model 4) macro of SPSS26.0 software (44). Thirdly, we investigated the moderating effect of gratitude on the indirect relationship between grit and depression by using the PROCESS (Model 14) macro of SPSS26.0 software (44).

## Results

### Preliminary Analysis

The results of Harman's single-factor test suggested that the variance for unrotated first factors was 26.69%, below the threshold of 40%, which indicated that there was no significant common method bias in the study (45).

Table 1 showed the descriptive statistics and means, standard deviations for all variables, including the bivariate correlations of grit, stressful life events, depression, life satisfaction. Grit was positively associated with life satisfaction (*r* = 0.426, *p* < 0.001) and negatively associated with depression (*r* = −0.167, *p* < 0.001). In addition, depression was negatively associated with life satisfaction (*r* = −0.236, *p* < 0.001). What's more, stressful life events were positively associated with depression (*r* = 0.595, *p* < 0.001).

**Table 1:** Descriptive statistics and correlations.

**Variable**	***M***	***SD***	**1**	**2**	**3**	**4**
1 Grit	3.967	0.591	1			
2 Stressful life events	1.267	0.939	0.086*	1		
3 Depression	1.086	0.744	−0.167***	0.595***	1	
4 Life satisfaction	4.552	1.2564	0.426***	−0.079*	−0.236***	1

n = 888; *p < 0.05, ***p < 0.001.

### Mediation Effect Analysis

We analyzed the data after adding age, gender, and grade as covariates. In Hypothesis 1, we assumed that depression would mediate the relationship between grit and life satisfaction. We tested this hypothesis with Model 4 of the PROCESS (44). As Table 2 showed, girt was positively associated with life satisfaction [β = 0.402, *SE* = 0.031, *p* < 0.001, 95% *CI* (0.342,0.462)] and negatively associated with depression [β = −0.173, *SE* = 0.033, *p* < 0.001, 95% *CI* (−0.238, −0.107)]. Depression was negatively associated with life satisfaction [β = −0.169, *SE* = 0.031, *p* < 0.001, 95% *CI* (−0.229, −0.110)]. The direct effect of grit on life satisfaction remained positive. Therefore, depression partially mediated the effect of grit on life satisfaction [indirect effect = 0.0292, *SE* = 0.009, 95% *CI*_boot_ = (0.135, 0.500)], accounting for 6.78% of the total effect. Therefore, Hypothesis 1 was supported.

**Table 2:** Testing the moderated mediation model.

**Predictor**	**Model 1 (Depression)**		**Model 2 (Life satisfaction)**		**Model 3 (Depression)**	
	**β**	***t***	**β**	***t***	**β**	***t***
Age	0.048	1.761	−0.030	−1.199	0.045	2.019*
Gender	−0.164	−2.452*	−0.010	−0.171	−0.058	−1.079
Grade	−0.084	−1.975*	−0.010	−0.270	−0.071	−2.100*
Grit	−0.173	−5.187***	0.402	13.140***	−0.111	−4.130***
Stress Life Events					0.562	20.709***
Depression			−0.169	−5.556***		
Grit × Stressful Life Events					0.107	3.878***
*R^2^*	0.039	0.213	0.381			
*F*	8.902***	47.669***	90.498***			

n = 888; *p < 0.05, **p < 0.01, ***p < 0.001.

### Moderated Mediation Effect Analysis

We analyzed the data after adding age, gender, and grade as covariates. In Hypothesis 2, we assumed that stressful life events would moderate the association between grit and depression. The PROCESS of the SPSS macro program was used to test the moderated mediation model and evaluate the moderating effect. The results of the moderated mediation test were shown in Model 3 of Table 2. The product (interaction term) of grit and stressful life events had a significant predictive effect on depression [β = 0.107, *SE* = 0.028, *p* < 0.001, 95% *CI* (0.053, 0.161)].

**Figure 2:**
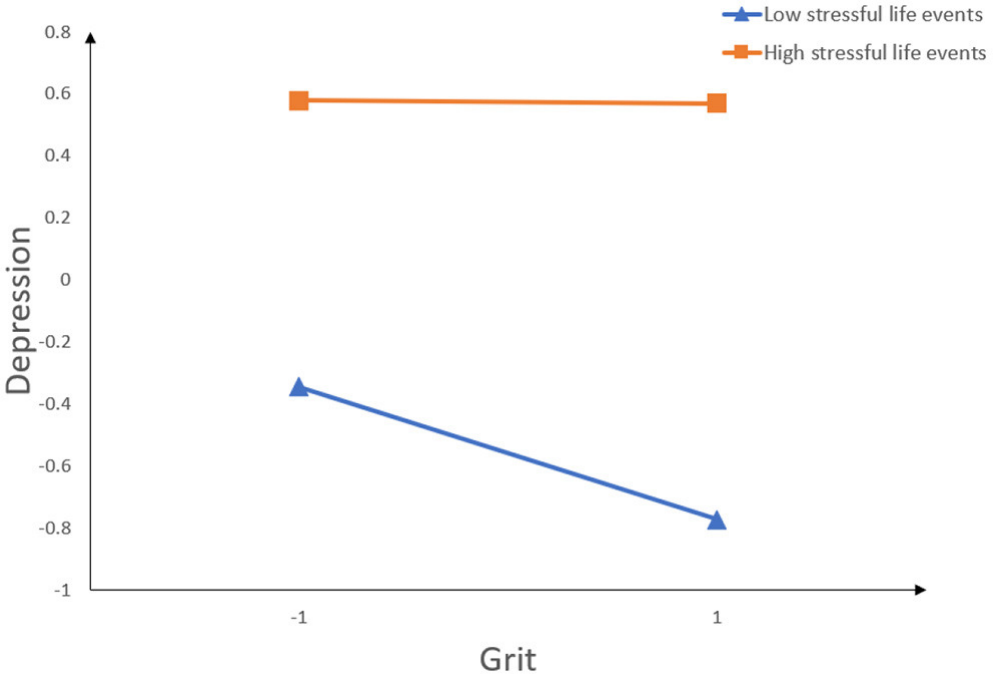
Interaction between grit and stressful life events on depression.

For description purposes, we plotted examined grit against depression, separately for low and high levels of gratitude. The interaction effect was visually plotted in Figure 2. Simple slope tests showed that for college students with highly stressful life events, grit was not significantly associated with depression, β_*simple*_ = −0.004, *t* = −0.102, *p* > 0.05, 95% *CI* = [−0.082, 0.074], and as for college students with low stressful life events, grit had significant effect on depression, β_*simple*_ = −0.218, *t* = −5.867, *p* < 0.001, 95% *CI* [−0.291,0.145]. In summary, these results indicated that stressful life events moderated indirect associations between grit and depression.

## Discussion

The COVID-19 has had a dramatic impact on individual's daily lives, particularly in the area of mental health. Life satisfaction is a vital indicator of individual's mental health, and it can measure the quality of an individual's life. People all over the world are eager to live a better life in the pursuit of happiness. At the same time, life satisfaction is not only an important indicator of individual adaptation to society but also an important symbol of social harmony. As an important social group, college students' life satisfaction during the recurrent outbreak of COVID-19 should be paid attention to, as well as the factors and mechanisms that affect college students' life satisfaction. The current study examined the relationship among grit, depression, stressful life events, and life satisfaction. The results of this study showed that depression played a partial mediating role in the relation between grit and college students' life satisfaction. Moreover, the relationship between grit and depression was further moderated by stressful life events. Our findings further contributed to the literature by testing a moderated mediation model, showing that depression acted as a mediator in the relationship between grit and life satisfaction. Moreover, the relationship between grit and depression was further moderated by stressful life events.

### The Relationship Between Grit and Life Satisfaction

This study examined the relationship between grit and life satisfaction and found that grit was significantly positively correlated with life satisfaction, which was consistent with previous research results (16, 18). Studies on grit have shown that the quality of grit can significantly predict individual achievement, in the academic field (12), the work field (14). At the same time, studies have also found that individuals with higher levels of grit have better mental health and can effectively cope with various emergencies caused by the COVID-19 (19). Generally speaking, grit, as an important personality trait in positive psychology, is also a psychological quality that can be cultivated, which has an important influence on individuals' physical and mental development. Combined with this study, life satisfaction can be improved and the negative impact of the epidemic on people can be weakened by cultivating individual grit during the recurrent outbreak of COVID-19.

### The Mediating Role of Depression

In this study, we added depression to explore the specific mechanism by which grit affected life satisfaction during the recurrent outbreak of COVID-19. The results showed that depression had a significant negative relation to college students' life satisfaction, which was consistent with the previous research results (27, 28). More importantly, this study found that depression was a bridge between grit and life satisfaction. Depression was found to partially mediate the relationship between grit and life satisfaction. In other words, the impact of grit on life satisfaction is not only direct, but also indirectly through depression. Here, depression played an important role as a bridge, which not only reflected the relationship with grit, but also reflected the relationship with life satisfaction, and answered “how” or “why” of grit would play a role in life satisfaction. Therefore, depression was an important internal cause of grit affecting college students' life satisfaction. Firstly, grit reduced the level of college students' depression, which was the same as the previous conclusion (26, 31). Grit as a stable positive personality trait can strongly influence an individual's attitude toward failure and misfortune (12). Specifically, Duckworth et al., (12) pointed out that in pursuit of long-term goals, individuals with high grit tend to remain perseverance and enthusiasm in the face of obstacles. Thus, grit can help an individual escape the belief that failure is inevitable in the pursuit of success, and ultimately resist the sense of difference that comes with high personal standards (29). In other words, for individuals with high grit, when facing negative results, there may be more optimistic attribution style (46). Therefore, high grit will reduce the level of depression. Previous studies have found that depression has a strong impact on individual life satisfaction (28, 47). In summary, during the period of recurrent outbreak of COVID-19, grit not only directly affected life satisfaction but also indirectly affected college students' life satisfaction by affecting college students' depression. This mediating role is of practical significance. On the one hand, it suggests that the impact of grit on life satisfaction is complex, on the other hand, the mediating effect provides a theoretical basis for improving life satisfaction of college students by reducing their depression.

### The Moderating Role of Stressful Life Events

From the perspective of the individual-environment interaction model (32), the influence of college students' factors (grit quality) and common environmental factors (stressful life events) on depression was comprehensively investigated. The results indicated that stressful life events moderated the relationship between grit and depression during the recurrent outbreak of COVID-19. To be specific, the relationship between grit and depression was weaker for college students with high stressful life events. However, the relationship between grit and depression was stronger for college students with low stressful life events. According to the stress vulnerability hypothesis, positive factors in individuals tend to lose their original protective effects in high-pressure environments (48). In the present study, the impact of stressful life events on individuals reduced their grit traits during the recurrent outbreak of COVID-19. According to the stress vulnerability hypothesis, grit loses its protective effect on depression when college students experience more stressful life events. Specifically, when individuals encounter more stressful life events, whether the level of individual grit is high or low, the level of depression of college students is at a high level. Our results showed that for college students who suffer less stressful life events, the higher the level of grit, the faster the level of depression decreases. Duckworth et al. (46) find that when facing negative results, individuals with high grit have a more optimistic attribution style. In particular, it should be noted that when stressful life events are high, the level of an individual's depression is very high regardless of whether the level of an individual's grit is high or low. In other words, people with high grit can benefit from reducing stressful life events, which supports the stress vulnerability hypothesis. This study shows that stressful life events play an important role in college students' depression. Therefore, this study also found that grit had a limited protective effect, and under high stressful life events, grit lost its protective effect. In order to reduce the depression problem of college students during the recurrent outbreak of COVID-19, it is necessary not only to cultivate the quality of grit but also should strive to reduce the pressure that college students may suffer, to promote the solution of college students' depression and improve their mental health.

In conclusion, grit affected college students' life satisfaction through depression during the recurrent outbreak of COVID-19, and the first half of this process was moderated by stressful life events. Schools and relevant social departments should create better living environment for college students, pay more attention to cultivating and improving individual grit, attach importance to students' depression and provide timely treatment, try to reduce the adverse effects of stressful life events that college students may encounter in their life, to improve the life satisfaction of college students.

### Limitations

There are some limitations in the current study that need to be noted. First of all, the cross-sectional design of this study makes it impossible to infer the causal relationship between variables, so experimental and longitudinal designs could be utilized in future research. Second, the self-reported questionnaire survey used in this study may be affected by social desirability, especially for variables with very high social desirability such as life satisfaction. In the future, measures with less social desirability effect, such as forced selection questionnaire, can be considered. Family socioeconomic status (SES) or household income may have influenced the results, and future research should collect the information about SES or household income and consider the effect of SES or household income on the results. Moreover, the subjects in this study were all college students, and the results should be tested in more groups in the future. Finally, COVID-19 is currently spreading around the world, and we can conduct further investigations in other countries seriously affected by COVID-19 to further validate the result in diverse samples.

Although there are some limitations, the contributions of the research are theoretical and practical. This study further extends previous research by examining the mediating role of depression and the moderating role of stressful life events. This study can inspire educators to pay attention to the cultivation of individuals' grit, promote their physical and mental health development, reduce the risk of depression and improve life satisfaction. Through this study, we found that stressful life events not only increase the risk of depression in college students but also reduce the level of life satisfaction in college students. Therefore, we should pay attention to improving the living environment of college students, and appropriately relieving pressure on college students, especially during this particular time of the recurrent outbreak of COVID-19.

## Conclusion

To sum up, the study was of great importance in exploring how grit was related to the Chinese college students' life satisfaction during the recurrent outbreak of COVID-19, even if further replication and extension were required. This study suggested that depression was an underlying mechanism through which grit was associated with life satisfaction. In addition, stressful life events moderated the relationship between grit and depression, and college students with high level of stressful life events had a weaker negative relationship between grit and depression during the recurrent outbreak of COVID-19.

## Data Availability Statement

The raw data supporting the conclusions of this article will be made available by the authors, without undue reservation.

## Ethics Statement

The studies involving human participants were reviewed and approved by school of Psychology, Jiangxi Normal University. Written informed consent to participate in this study was provided by the participants' legal guardian/next of kin. Written informed consent was obtained from the individual(s), and minor(s)' legal guardian/next of kin, for the publication of any potentially identifiable images or data included in this article.

## Author Contributions

HL: conceptualization, investigation, writing–original draft, visualization, and revised manuscript. ZY: conceptualization, writing–original draft. BY: conceptualization, methodology, investigation, statistical analysis, data curation, visualization. QY: project administration, and funding acquisition. All authors read and approved the final manuscript.

## Funding

This study was supported by the National Natural Science Foundation of China (72164018), National Social Science Fund Project (BFA200065), Jiangxi Social Science Foundation Project (21JY13), Jiangxi' Key Research Base Project of Humanities and Social Sciences (JD20068) and Science and Technology Research Project of Jiangxi' Department of Education (GJJ200306).

## Conflict of Interest

The authors declare that the research was conducted in the absence of any commercial or financial relationships that could be construed as a potential conflict of interest.

## Publisher's Note

All claims expressed in this article are solely those of the authors and do not necessarily represent those of their affiliated organizations, or those of the publisher, the editors and the reviewers. Any product that may be evaluated in this article, or claim that may be made by its manufacturer, is not guaranteed or endorsed by the publisher.
